# Is the right to disconnect a testable organizational hypothesis for physiological recovery? A comment on after-hours ICT use

**DOI:** 10.1093/joccuh/uiag045

**Published:** 2026-08-10

**Authors:** Carlo Aresti

**Affiliations:** Specialist in Occupational Medicine and Specialist in Forensic Medicine, Independent Researcher, Paris, France

**Keywords:** right to disconnect, information and communication technology, heart rate variability, cortisol awakening response, workplace telepressure, occupational health

Dear Editor,

We read with great interest the studies by Kubo et al^1^ and Ikeda et al^2^ on after-hours use of information and communication technology (ICT) among information technology (IT) employees. Kubo et al found that after-hours ICT use was associated with reduced psychological detachment, increased rumination, and poorer sleep quality, and that shorter off-job time, rather than ICT use itself, was independently associated with a higher cortisol awakening response (CAR).^1^ Ikeda et al similarly found that longer after-hours electronic communication was associated with worse fatigue, worse depression, and lower psychomotor vigilance before bedtime.^2^ We commend the authors for these carefully conducted studies. Read alongside the wider occupational stress-physiology literature, we believe these findings justify prospective studies that test whether the right to disconnect (RTD)—the entitlement of workers to refrain from work-related electronic communication outside contracted hours—affects biological recovery, rather than assuming that it does. This synthesis is intended as hypothesis-generating rather than confirmatory, drawing together literatures on ICT use, workplace psychology, and stress physiology that have not previously been considered jointly. A plausible biological pathway is outlined below.

After-hours ICT use appears to act mainly through workplace telepressure, the urge to respond promptly to work messages, which in turn fuels work-related rumination and impairs psychological detachment. Both plausibly disturb 2 recovery-relevant biomarkers, nocturnal heart rate variability (HRV) and CAR, though the evidence for each link is uneven. The telepressure-to-rumination step has recent field support in an educator sample.^3^ The telepressure-to-detachment link is the best supported, evidenced by a meta-analysis of 91 samples (38 124 employees) linking detachment to reduced exhaustion and better sleep.^4^ The rumination-to-HRV link has direct field support.^5^ The CAR segment is weaker, since Kubo et al tied it to off-job duration rather than ICT use itself.

Two considerations qualify this pathway. Of course, nocturnal HRV and CAR are shaped by more than after-hours ICT use: chronotype, shift schedule, workload, sleep debt, and nonwork stressors all plausibly contribute, and the cited observational studies could only partially account for them. Separately, the 2 cohort studies providing the most direct occupational evidence share an overlapping research group and IT-sector samples,^1^^,^^2^ so independent replication in other sectors and countries is needed. Most importantly, no published trial has evaluated RTD implementation itself against biological recovery endpoints.

Given these constraints, it may be informative to pursue incremental extensions of the existing cohorts rather than a new trial. Future analyses of the Kubo et al dataset could examine whether shorter off-job time and after-hours ICT use independently predict CAR, or whether one factor accounts for the other. This distinction has practical relevance: if ICT use itself drives the signal, organizational policies could target message volume and response expectations specifically, rather than relying on blanket restrictions on off-job duration, which are typically harder to enforce and monitor in practice. Should either cohort be extended or repeated, adding a validated ambulatory HRV measure alongside the existing CAR sampling would allow the telepressure-to-detachment link to be tested against a second recovery biomarker, without requiring new trial infrastructure. We would welcome the authors’ views on the feasibility of either extension.

In conclusion, we congratulate Kubo et al and Ikeda et al on findings that, in our view, extend well beyond their original occupational focus. We hope these observations encourage further analyses of the existing data, and eventually prospective evaluation, of the RTD against biological recovery endpoints.

## Data Availability

No new data were generated or analysed in support of this article.
